# EB1 Recognizes the Nucleotide State of Tubulin in the Microtubule Lattice

**DOI:** 10.1371/journal.pone.0007585

**Published:** 2009-10-23

**Authors:** Marija Zanic, Jeffrey H. Stear, Anthony A. Hyman, Jonathon Howard

**Affiliations:** Max Planck Institute of Molecular Cell Biology and Genetics, Dresden, Germany; University of Birmingham, United Kingdom

## Abstract

Plus-end-tracking proteins (+TIPs) are localized at the fast-growing, or plus end, of microtubules, and link microtubule ends to cellular structures. One of the best studied +TIPs is EB1, which forms comet-like structures at the tips of growing microtubules. The molecular mechanisms by which EB1 recognizes and tracks growing microtubule ends are largely unknown. However, one clue is that EB1 can bind directly to a microtubule end in the absence of other proteins. Here we use an *in vitro* assay for dynamic microtubule growth with two-color total-internal-reflection-fluorescence imaging to investigate binding of mammalian EB1 to both stabilized and dynamic microtubules. We find that under conditions of microtubule growth, EB1 not only tip tracks, as previously shown, but also preferentially recognizes the GMPCPP microtubule lattice as opposed to the GDP lattice. The interaction of EB1 with the GMPCPP microtubule lattice depends on the E-hook of tubulin, as well as the amount of salt in solution. The ability to distinguish different nucleotide states of tubulin in microtubule lattice may contribute to the end-tracking mechanism of EB1.

## Introduction

Microtubules are dynamic cytoskeletal polymers that play important roles in intracellular transport, mitosis and cell polarity [1]. Defined by their localization at microtubule fast-growing (‘plus’) end, plus-end-binding proteins (+TIPs) regulate a variety of dynamic processes associated with this microtubule end and link microtubule ends to cellular structures such as kinetochores and the cell cortex [2].

EB1 is a highly conserved +TIP, which locates to the ends of polymerizing microtubules *in vivo* [3]. It directly interacts with many other +TIPs and is therefore potentially central to the assembly of +TIP complexes at microtubule ends [4]. Recent studies [5]–[7] have established that EB1 autonomously localizes at growing microtubule ends *in vitro* and plays a role in the plus-end localization of other +TIPs. The same studies show that the mechanism of EB1 tip-tracking most likely relies on the recognition of a structural feature at the microtubule end. However, the specifics of this structural feature are not known and several possibilities remain open.

One possible feature that EB1 might be recognizing at the plus end is the GTP cap. GTP-tubulin undergoes the process of GTP-hydrolysis as it becomes incorporated in the microtubule lattice, resulting in a GDP-tubulin lattice, which is more unstable than the GTP-tubulin lattice [1], [8]. The growing plus end is thought to maintain a GTP- or GDP-Pi- tubulin cap [8], [9], which would help stabilize the microtubule lattice, and could be the feature recognized by EB1. Another possibility is that EB1 binds to specific tubulin sites that are exposed only at the growing ends, or to a particular curved tubulin conformation specific to the ends (for example open sheets) [10].

In this study, we use an *in vitro* assay for microtubule dynamics with two-color total-internal-reflection-fluorescence (TIRF) microscopy to show that mammalian EB1 autonomously tip-tracks growing microtubule ends *in vitro*. Furthermore, using GMPCPP, a slowly hydrolyzable analogue of GTP, we find that EB1 preferentially binds GMPCPP- over GDP-tubulin lattice. This indicates that the mechanism of EB1 plus-end tracking may rely on its recognition of the nucleotide state of the growing end.

## Results

### Human EB1-GFP is a +TIP *in vitro*

To confirm recent reports [7] that human EB1 is a plus-tip-tracking protein *in vitro*, we visualized EB1 interacting with microtubules polymerizing from stabilized seeds using two-color TIRF microscopy. Seeds were made using 11% rhodamine-labeled and 17% biotinylated GMPCPP-tubulin and bound to silanized coverslips coated with neutravidin [11]. GMPCPP is a slowly hydrolyzable analogue of GTP thought to trap tubulin in a GTP-like state, leading to highly stable microtubules [12]. A reaction mix containing 12 µM free tubulin (25% rhodamine-labeled), 1 mM GTP and 50 nM C-terminus GFP-tagged EB1 in Imaging Buffer (for details see Methods) was perfused into the chamber. Microtubule growth by extension from existing GMPCCP seeds was observed by TIRF visualization of polymerizing rhodamine-labeled tubulin.

**Figure 1:**
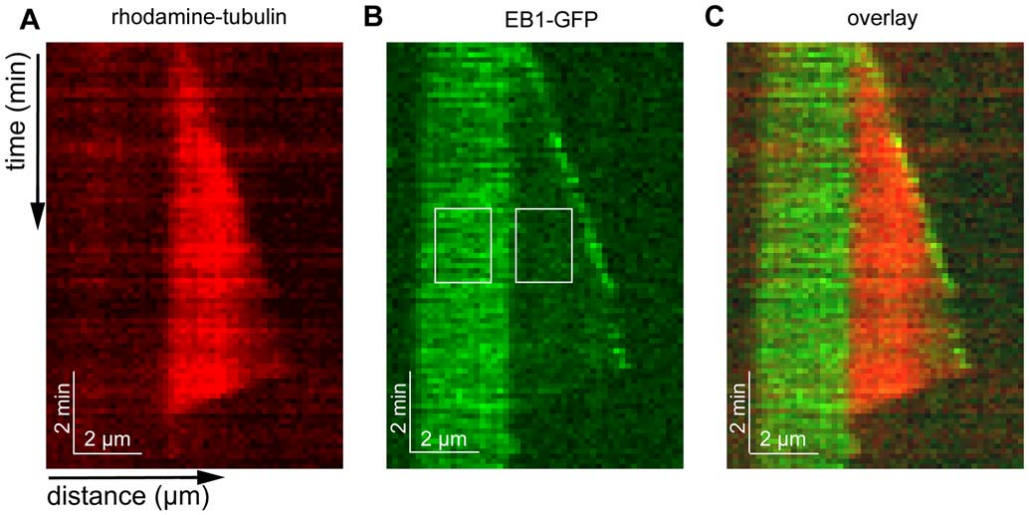
EB1-GFP prefers GMPCPP- over GDP-tubulin microtubules. A Kymograph of microtubule growth with rhodamine-labeled tubulin (12 µM, red), taken from time-lapse microscopy. B Kymograph of EB1-GFP (50 nM, green) showing tip-tracking, as well as preferential binding to microtubule seeds over extensions. The average fluorescence intensities within the marked rectangular areas are: seed-background: 62.6±4.7 AU (SE, *n* = 30), and extension-background: 12.8±2.8 AU (SE, *n* = 30). C Overlay of A and B.

Time-lapse TIRF visualization of GFP-tagged EB1 showed bright foci of EB1-GFP moving away from microtubule seeds at locations corresponding to the growing microtubule ends, confirming that EB1-GFP is capable of tip-tracking growing microtubule ends without the need of any protein binding partners (Figure 1). Tip-tracking was observed at both the plus and minus ends of growing microtubules (Movie S1, Figure S1), but no end localization was seen for microtubules that were not growing. Bright EB1-GFP foci were not observed at the microtubule ends during periods of microtubule depolymerization. The above findings confirm previous results that the tip-tracking of EB1 relies on the process of microtubule growth.

### EB1-GFP binds lattice, with a preference for GMPCPP- over GDP-tubulin

We also observed that EB1-GFP binds the microtubule lattice, and that this binding is enhanced on GMPCPP-tubulin microtubule lattice compared to GDP-tubulin extensions. With extensions grown using 12 µM free tubulin in the presence of 50 nM EB1-GFP in Imaging Buffer, we observed lattice binding along the whole length of microtubules, with the average GFP fluorescence intensity on the microtubule seeds being about five times higher than on the newly grown extensions, and comparable to that of the growing end (Figure 1).

Enhanced binding on GMPCPP microtubules was observed in a number of different conditions. GMPCPP microtubule seeds were grown with different rhodamine-labeling ratios and were either biotinylated and bound to the surface using biotin-neutravidin links, or non-biotinylated GMPCPP seeds were immobilized using anti-rhodamine antibodies. Microtubule extensions were grown by addition of free tubulin in the concentration range of 10 µM to 24 µM; tubulin was either rhodamine-labeled, Alexa 546-labeled or unlabeled. In all cases we found higher EB1-GFP fluorescence intensity on the GMPCPP microtubule lattice compared to the extension. Because the lattice of microtubule extensions grown with GTP is known to consist of GDP-tubulin [8], these findings suggest that EB1-GFP can distinguish between the different nucleotide states of tubulin in seeds and extensions.

**Figure 2:**
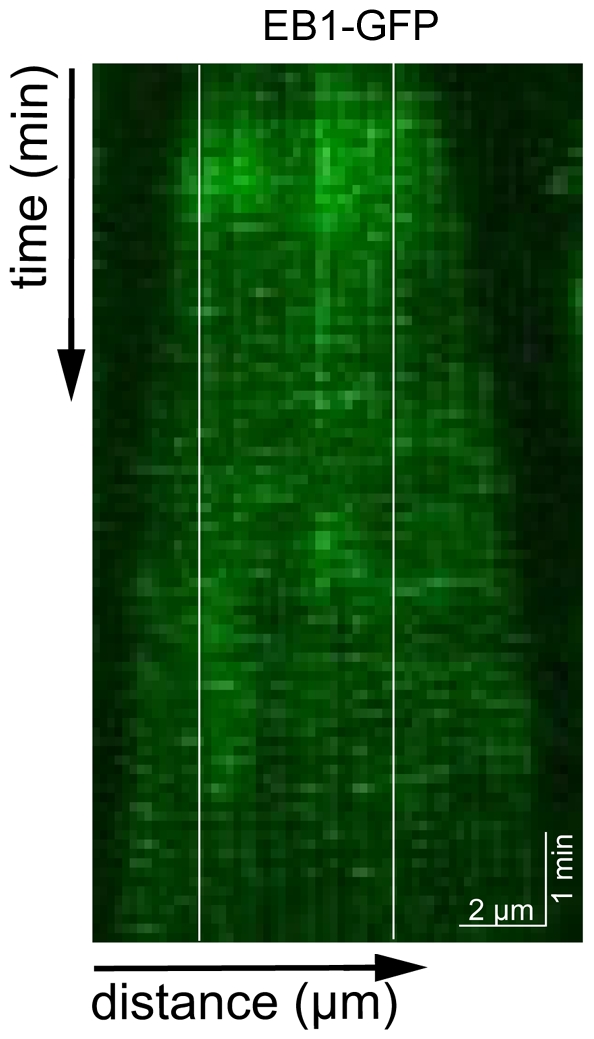
EB1-GFP binds GMPCPP-tubulin in the seed and the extension. Kymograph of EB1-GFP (50 nM) binding growing microtubule lattice (6 µM unlabeled tubulin with GMPCPP). White lines indicate the extension of the microtubule seed.

To test whether EB1 is specifically recognizing the nucleotide state of the lattice (rather than some other property such as its age), we repeated the experiment with microtubule extensions grown from unlabeled GMPCPP-tubulin. In this case we observed uniform binding of EB1-GFP throughout the microtubule lattice, with no preferential binding to seeds, extensions or ends (Figure 2). We do not attribute the absence of EB1 tip-tracking in GMPCPP tubulin to the low microtubule growth rate (and therefore small comet), since EB1 tip-tracking in GTP tubulin was observed at comparably low growth rates. This experiment also served as a control for the potential surface effects and effects of rhodamine-labeling, since it showed that EB1 bound equally well to the surface-bound rhodamine-labeled seeds as to the unlabeled non surface-bound extensions.

### EB1-GFP binds the microtubule lattice through an electrostatic interaction with the E-hook of tubulin

**Figure 3:**
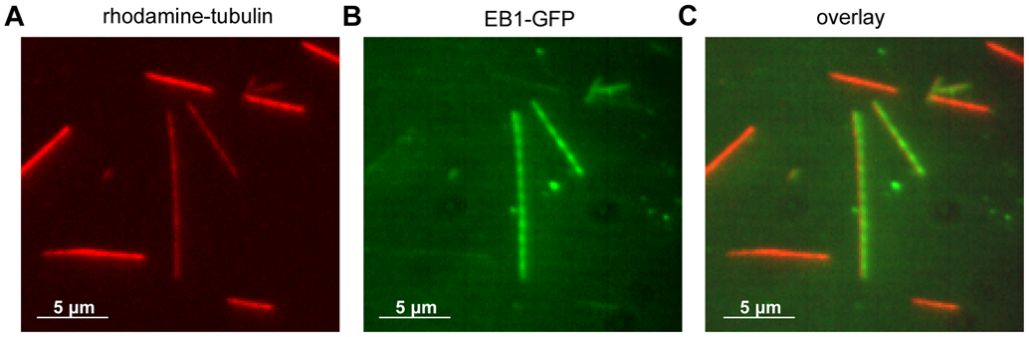
EB1-GFP lattice binding is mediated by the tubulin E-hook. A Subtilisin-digested microtubules (bright) along with normal untreated microtubules (dim). B EB1-GFP (12.5 nM) lattice binding suppressed on subtilisin-digested microtubules. C Overlay of A and B.

Lattice binding of microtubule-associated proteins such as XMAP215 [13] and the depolymerizing kinesin-13 MCAK [11] is mediated by the E-hook of tubulin [14]. To determine whether EB1 also binds via the E-hook, we treated microtubules with subtilisin, a bacterial protease which specifically removes the tubulin E-hook. We bound both subtilisin-treated and untreated control GMPCPP microtubules in the same flow-cell, distinguished by different rhodamine-labeling ratios. Upon introducing EB1-GFP in the flow-cell, we found that EB1-GFP lattice binding is strongly reduced on the lattice of subtilisin-digested microtubules (Figure 3).

**Figure 4:**
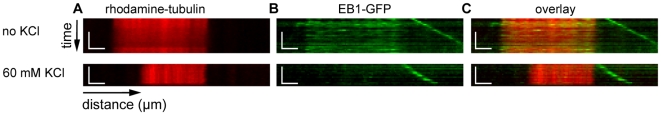
EB1-GFP tip-tracking persists with higher salt. A Kymographs of rhodamine-labeled microtubule seeds. B EB1-GFP (50 nM) binding to growing microtubules (24 µM unlabeled tubulin). C Overlay of A and B. Top row: EB1-GFP tip-tracks and preferentially binds to GMPCPP seeds in Imaging Buffer with no additional salt. Bottom row: Preferential binding to GMPCPP seeds is abolished, but tip-tracking persists when EB1-GFP introduced in Imaging Buffer with an additional 60 mM KCl. Scale bars: 2 µm (horizontal) and 1 min (vertical).

To further investigate the electrostatic nature of this interaction, we examined binding of EB1-GFP to microtubule lattice in varying buffer salt conditions (Figure S2). We found that EB1-GFP lattice binding is antagonized by high salt; addition of 40 mM or more KCl to the Imaging Buffer results in a strong reduction in EB1-GFP lattice binding. However, we also found that enhanced binding of EB1-GFP on growing microtubule ends persists in higher salt conditions (Figure 4). This finding is consistent with previous *in vitro* studies [5], [6] in which enhanced EB1 binding to the GMPCPP microtubule lattice was not observed, since these previous studies were performed under higher salt conditions.

## Discussion

In this paper, we show that EB1 can distinguish the nucleotide state of tubulin in the microtubule lattice. Specifically, EB1 binds more strongly to the GMPCPP microtubule lattice than to the GDP microtubule lattice. This ability to recognize the nucleotide state of tubulin in the lattice could play a role in the microtubule end-tracking mechanism of EB1, since the growing microtubule end is thought to maintain a GTP-tubulin cap (and GMPCPP is an analogue of GTP). Further support for such an end-recognition mechanism is our observation of no enhanced EB1 binding to microtubule ends growing in GMPCPP tubulin. This latter observation is consistent with a recent report that EB1-dependent CLIP170 microtubule end-tracking is not seen when GMPCPP tubulin is used [7]. Thus, several observations suggest that EB1 may be recognizing the nucleotide state of the tubulin at the growing end of the microtubule.

Does EB1 also recognize a non-nucleotide dependent structure specific to the GTP-tubulin growing end? Such a possibility would explain our finding that increasing the amount of salt in solution suppresses GMPCPP lattice binding, while tip-tracking of growing GTP-tubulin ends is maintained. However, an alternative explanation for the preferential end-binding in GTP tubulin over GMPCPP-seed binding is that GMPCPP is not an exact analogue of GTP: EB1 might have a higher affinity for tubulin in the GTP or GDP-Pi state (at the growing end), compared to the GMPCPP state (in the seed). Thus, while our evidence supports a nucleotide-recognition mechanism, it does not rule out the possibility of an additional non-nucleotide-dependent mechanism.

It has recently been suggested that EB1 induces changes in the lattice structure of microtubules grown in its presence [15], which could also provide a distinguishing feature between the microtubule seeds and extensions in our experiments. If this were the case, one would expect enhanced binding on the extensions rather than the seeds, because the extensions and not the seeds are grown in the presence of EB1. However, we never observed stronger binding on the extensions.

In addition to the effect of nucleotide state, we find that EB1 lattice binding also depends on the E-hook of tubulin. This is interesting because the GTP binding site on tubulin is concealed within the interdimer surface, whereas the E-hook is an unstructured peptide located on the outer surface of the microtubule. One possibility is that EB1 uses both the E-hook as well as the GTP dimer conformation to preferentially bind to the growing end of the microtubule. An alternative possibility is that the GTP-state of tubulin modulates the accessibility of the E-hook for EB1 binding.

We do not know how many microtubule-associated proteins recognize the nucleotide state of the microtubule. A previous study reported GTP-tubulin lattice as a preferred binding partner of the 10-protein Dam1 ring complex in budding yeast [16]. It is interesting that reconstituted kinetochores, which use the passenger proteins Bir1 and Sli15 to link to microtubules [17], can similarly distinguish the GTP from the GDP lattice [18]. Future work will be required to establish whether these reconstituted kinetochores were recognizing the GTP state of the lattice through EB1, or through other proteins.

## Materials and Methods

### Protein preparation

Tubulin was purified from porcine brains and labeled according to the standard protocols [19], [20]. GMPCPP microtubules were prepared as previously described [21]. Subtilisin treatment of microtubules was performed as previously described [11].

The coding region of His-EB1 OmicsLink Expression Clone (GeneCopoeia) was modified by addition of a C-terminal GFP tag and cloned into pETMM-11 vector. The recombinant fusion protein was expressed in E. Coli (Rosetta Cells) and purified using a Ni Sepharose column (HisTrap HP, GE Healthcare). Protein concentration was determined using a Bradford assay and absorbance at λ = 280 nm.

### Assay Conditions

Silanization of cover glasses and preparation of flow-cells was previously described [11]. The assay protocol for immobilization of microtubules in a flow-cell was previously described [13]. The Imaging Buffer consisted of BRB80 supplemented with 40 mM glucose, 40 µg/ml glucose-oxidase, 16 µg/ml catalase, 0.1 mg/ml casein and 1% β-mercaptoethanol. For the experiments involving microtubule growth, an objective heater (Zeiss) was used to warm the sample to 35°C.

### Imaging

The imaging setup utilizing total-internal-reflection fluorescence was previously described [11]. Images were collected with Andor iXon camera on a Zeiss Axiovert 200 M microscope using a Zeiss 100X/1.45 α FLUAR objective. Standard filter sets were used to visualize GFP and TAMRA fluorescence.

## Supporting Information

Figure S1 EB1-GFP tip-tracking growing microtubule plus- and minus-ends. Kymograph from Movie S1 showing EB1-GFP (50 nM) tip-tracking on both ends. Scale bars 1 µm (horizontal) and 30 sec (vertical).
(0.92 MB TIF)

Figure S2 EB1-GFP lattice binding is antagonized by high salt. The effect of the ionic strength of the buffer examined by successive perfusion of EB1-GFP in different salt conditions in the same flow cell. Top row: rhodamine-labeled microtubules; middle row: EB1-GFP (25 nM); bottom row: overlay. A Imaging Buffer with no additional KCl. B Imaging Buffer with 100 mM KCl; lattice binding suppressed. C Imaging Buffer with no additional KCl; lattice binding recovered. D Imaging Buffer with 10 mM KCl. E Imaging Buffer with 40 mM KCl.
(5.75 MB TIF)

Movie S1 EB1-GFP tip-tracking growing microtubule plus- and minus-ends. Time-lapse of GMPCPP microtubule seeds (red, imaged by EPI fluorescence) with 24 µM unlabeled tubulin, 50 nM EB1-GFP (green, imaged by TIRF) in Imaging Buffer (see Methods) with additional 60 mM KCl. Images were taken at 5 second intervals. Video-playback is 50× times real time. Area size is 45 µm×31 µm.
(2.08 MB MOV)
