# Supplementary figures and images for: EB1 Recognizes the Nucleotide State of Tubulin in the Microtubule Lattice
Source: PLoS One. 2009 Oct 23;4(10):e7585. doi: 10.1371/journal.pone.0007585 (PMC2761489; doi:10.1371/journal.pone.0007585)

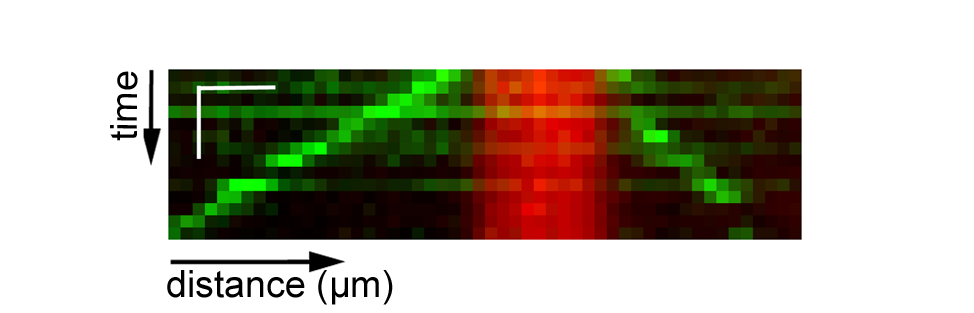

Supplement: Figure S1 — EB1-GFP tip-tracking growing microtubule plus- and minus-ends. Kymograph from Movie S1 showing EB1-GFP (50 nM) tip-tracking on both ends. Scale bars 1 µm (horizontal) and 30 sec (vertical). (0.92 MB TIF) [file pone.0007585.s001.tif]

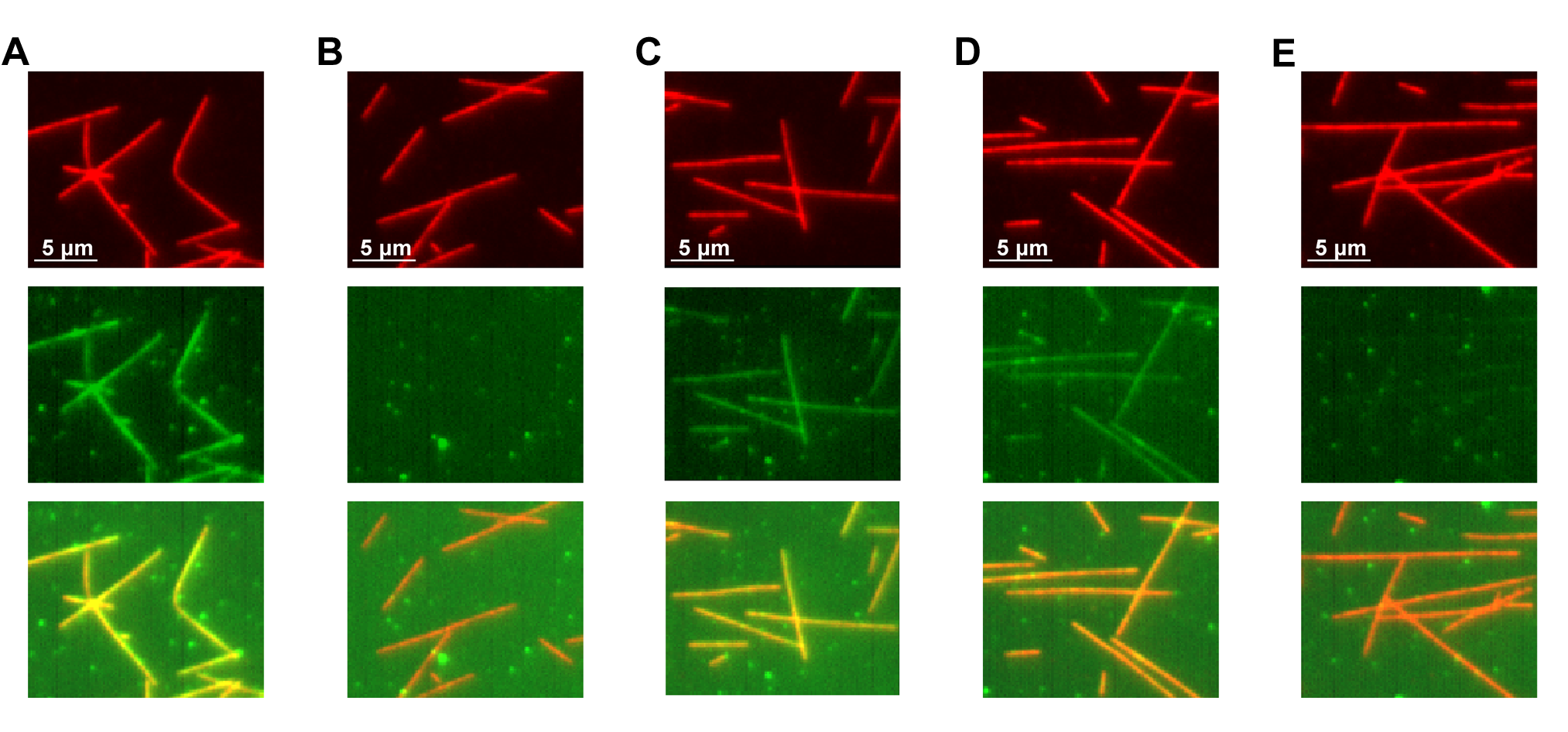

Supplement: Figure S2 — EB1-GFP lattice binding is antagonized by high salt. The effect of the ionic strength of the buffer examined by successive perfusion of EB1-GFP in different salt conditions in the same flow cell. Top row: rhodamine-labeled microtubules; middle row: EB1-GFP (25 nM); bottom row: overlay. A Imaging Buffer with no additional KCl. B Imaging Buffer with 100 mM KCl; lattice binding suppressed. C Imaging Buffer with no additional KCl; lattice binding recovered. D Imaging Buffer with 10 mM KCl. E Imaging Buffer with 40 mM KCl. (5.75 MB TIF) [file pone.0007585.s002.tif]
